# Potential Therapeutic Targeting of Coronavirus Spike Glycoprotein Priming

**DOI:** 10.3390/molecules25102424

**Published:** 2020-05-22

**Authors:** Elisa Barile, Carlo Baggio, Luca Gambini, Sergey A. Shiryaev, Alex Y. Strongin, Maurizio Pellecchia

**Affiliations:** 1 Division of Biomedical Sciences, School of Medicine, University of California Riverside, Riverside, CA 92521, USA; elisabarile@gmail.com (E.B.); carlo.baggio@medsch.ucr.edu (C.B.); lucaga@ucr.edu (L.G.); 2 Sanford Burnham Prebys Medical Discovery Institute, La Jolla, CA 92037, USA; shiryaev@sbpdiscovery.org (S.A.S.); strongin@sbpdiscovery.org (A.Y.S.)

**Keywords:** COVID19, SARS-COV2, Anthrax toxin, protecting antigen, furin, TMPRSS2

## Abstract

Processing of certain viral proteins and bacterial toxins by host serine proteases is a frequent and critical step in virulence. The coronavirus spike glycoprotein contains three (S1, S2, and S2′) cleavage sites that are processed by human host proteases. The exact nature of these cleavage sites, and their respective processing proteases, can determine whether the virus can cross species and the level of pathogenicity. Recent comparisons of the genomes of the highly pathogenic SARS-CoV2 and MERS-CoV, with less pathogenic strains (e.g., Bat-RaTG13, the bat homologue of SARS-CoV2) identified possible mutations in the receptor binding domain and in the S1 and S2′ cleavage sites of their spike glycoprotein. However, there remains some confusion on the relative roles of the possible serine proteases involved for priming. Using anthrax toxin as a model system, we show that in vivo inhibition of priming by pan-active serine protease inhibitors can be effective at suppressing toxicity. Hence, our studies should encourage further efforts in developing either pan-serine protease inhibitors or inhibitor cocktails to target SARS-CoV2 and potentially ward off future pandemics that could develop because of additional mutations in the S-protein priming sequence in coronaviruses.

## 1. Introduction

**Figure 1:**
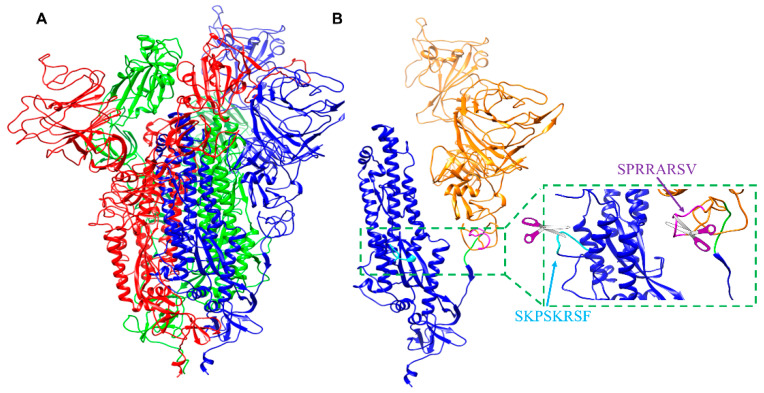
Model of the SARS-CoV2 spike glycoprotein and processing sites. (**A**) Molecular model of the trimeric (red, blue, and green) S-glycoprotein from SARS-CoV2. The model was obtained by Swiss3D model repository and based on the experimentally derived structure of the protein (PDB ID 6VSB) [1]. (**B**) Molecular model of the S-glycoprotein as in (**A**) but only one chain is displayed. The S1 N-terminal subunit is shown in orange, while the S2 C-terminal subunit is depicted in blue. The S1 furin cleavage site, the S2 cleavage site, and second S2′ cleavage site, are highlighted in magenta, green, and cyan respectively.

The outer surface of coronaviruses contains a critical transmembrane spike glycoprotein that is essential for entry of viral particles into host cells. This viral glycoprotein possesses a trimeric structure, which gives the virus its typical crown-like halo (Figure 1A). This outer protein contains domains and structural motifs that are essential for binding to host cells and for viral fusion. Two major subunits (S1 and S2) need to be processed by host cell proteases to enable conformational rearrangement of the C-terminal domain and exposure of the epitopes that allow the virus to enter and subsequently egress from host cells (Figure 1B) [1,2]. Hence, recent studies suggested that impairing the spike glycoprotein processing represents a viable therapeutic strategy [3,4]. There are three proteolytic cleavage sites (S1, S2, and S2′; Figure 1B) in the spike glycoprotein. The sequence of these sites can determine whether the virus can cross species, for example from bats or camels to humans [5,6,7,8]. The cleavage site (S2) of sequence ATY↓MS (the arrow indicates the cleavage site) is likely cleaved by cathepsin L (Figure 1B) [8]. Because this site is conserved among coronaviruses, its cleavage cannot explain differences in pathogenicity among them [3].

On the contrary, and unlike less virulent coronavirus strains, the SARS-CoV2 glycoprotein presents the S1 cleavage of sequence SP**R**RA**R**↓**SV** (Table 1; consensus residues are depicted in bold characters), which represents a consensus furin recognition motif [3]. Furin and related proprotein convertases (PC2, PC1/3, PC4, PACE4, PC5/6, and PC7) are specialized serine endoproteases, which cleave R-X-(R/K/X)-R↓(S)(V/A/L) multibasic motifs [9,10,11]. The highly pathogenic MERS-CoV coronavirus also contains a putative furin cleavage S1 site [2,12] (Table 1). On the contrary, less pathogenic strains such as the SARS coronavirus (SARS-CoV) and the bat coronavirus strains (Bat-RaTG13, Bat-ZXC21, or Bat-ZC45) possess the S1 sequence S(L/I)L**R**↓**S**T, which cannot be readily cleaved by furin. For these sites, the membrane trypsin-like serine protease, TMPRSS2, has been identified as a possible major priming protease [8]. This observation suggests that furin may be essential for the viral entry and/or egress in highly pathogenic strains [2,3].

However, earlier studies indicated that furin was dispensable for the MERS-CoV entry, while TMPSSR2 was necessary and sufficient for viral entry [13]. A more recent study with SARS-CoV2 corroborated these findings [4]. In particular, pharmacological inhibition of TMPRSS2 by camostat mesylate, a covalent inhibitor, attenuated the entry of SARS-CoV2 surrogate viral particles into human cells, albeit only partially and at relevantly high (10–50 μM) concentrations [4]. Here, we closely analyzed the coronavirus cleavage sequences to delineate possible parameters that may confer increased virulence and pathogenicity to SARS-CoV2. In contrast to other coronaviruses, the S1 SARS-CoV2 site presents the peculiar property of being a substrate for both furin and TMPRSS2. Furin is involved in numerous pathogenic processes including not only viral propagation but also bacterial toxin activation. In anthrax toxin, for example, furin plays an essential role in the cleavage of anthrax protective antigen (PA), representing a necessary step for the entry of anthrax toxin into macrophages. Anthrax toxin PA protein (83 kDa) is secreted by the bacterium, binds to anthrax toxin receptor (ATR) on host cell membranes and is then cleaved by cell-surface furin to generate a cell-associated 63 kDa PA and a free 20 kDa PA [14,15,16,17,18,19,20,21,22,23,24,25]. The cell-associated PA molecule heptamerizes, forming a membrane channel that allows entry of lethal factor (LF) toxin into the host-cell cytoplasm, resulting in shock and eventual death. If furin is absent or inactive, the toxin fails to assemble and therefore is not lethal. Interestingly, the S1 SARS-CoV2 site is homologous to the processing site of the anthrax toxin PA protein, which can also be processed by TMPRSS2-like proteases and furin (Table 1). Moreover, similar to LF/PA anthrax toxin, SARS-CoV infects macrophages, as well as the airway epithelium [26]. Hence, we monitored the ability of compound **1** to protect RAW macrophages from LF/PA-induced cell death. The agent protected RAW macrophages in a dose-dependent manner with EC_50_ in the low micromolar range indicating a robust cellular inhibition of various possible PA-activating serine proteases, including furin and, potentially, also TMPRSS2. Finally, using an in vivo anthrax toxemia model, we probed whether systemic administration of serine protease inhibitors is potentially a viable therapeutic strategy.

## 2. Results

### 2.1. Sequence Analyses of Priming Sequences in Spike Glycoproteins

The S1 and S2′ cleavage sequence of selected coronavirus strains are shown in Table 1 [3]. Cleavage preferences by furin and furin-like serine proteases have been well characterized. Because furin cleaves the **R**-X-(R/K/X)-**R**↓(**S**)(V/A/L) multibasic motif [9,10,11], the prediction of furin cleavage sequences is possible with a high level of confidence [27]. Using the ProP server (http://www.cbs.dtu.dk/services/ProP), sequences can be classified for their propensity to be cleaved by furin [27]. A score <0.5 indicates sequences that are predicted to not be cleaved by furin, while scores between 0.5 and 1 indicate highly likely furin cleavage sites (Table 1) [27]. A rank-ordering that is in agreement with the ProP score was also experimentally verified by monitoring 1D ^1^H NMR spectral changes of selected peptides representing the cleavage motifs reported in Table 1, when measured over time in presence of a catalytic amount of furin (Figures S11 and S12; Supplementary Materials). 

**Figure 2:**
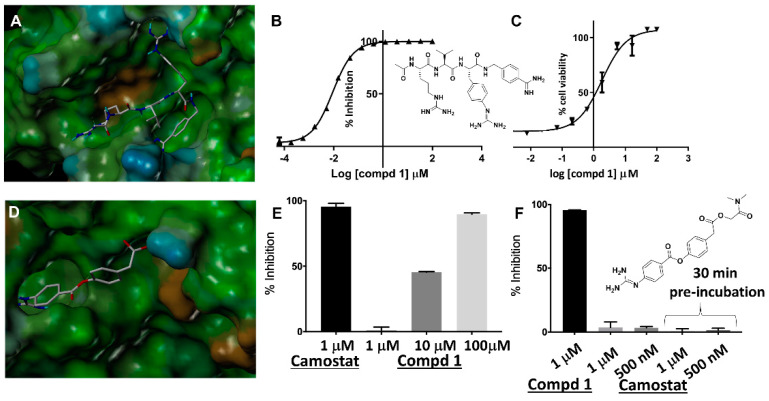
Characterization of the protease inhibitors. (**A**) Docked geometry of compound **1** into the 3D structure of furin. The docked pose was obtained based on the X-ray structure of similar agents in complex with furin (PDB-ID 4OMC) [30]. The docked geometry was obtained based on the structure of the complex using Sybyl (Cetara, St. Louis) and visualized using MOLCAD (surface representation color-coded according to lipophilic potential: brown more lipophilic; cyan less lipophilic). (**B**) Chemical structure of compound **1** and dose response curve for inhibition of furin by compound **1**. (**C**) Dose response curves for the protection of RAW macrophages by lethal factor/protective antigen (LF/PA) apoptosis by compound **1**. (**D**) Docked geometry of camostat in a modeled structure of TMPRSS2 catalytic domain (obtained via Swiss3D model and bovine trypsin as template, PDB ID 3MFJ). (**E**) Inhibition of bovine trypsin by camostat, as a reference, and compound **1** at the indicated conditions. (**F**) Chemical structure of camostat and inhibition of furin by compound **1** and camostat at the indicated conditions.

The pathogenic coronaviruses appear to contain a furin consensus motif in either the S1 or S2′ sites (Table 1). However, previous data in MERS-CoV (also containing putative furin cleavage sites in both S1 and S2′, Table 1) [12,13] and SARS-CoV2 indicate that furin is more dispensable than TMPRSS2 for viral entry [4,28]. While cleavage preferences of TMPRSS2 and other TMPRs are less defined compared to furin, their common consensus motifs follow the order **R↓K** > **R↓R** >> **R↓X** [29]. These simple motifs are the result of the small size of the TMPRSS2 binding site, nearly identical to that of trypsin, compared with the larger binding pocket of furin (Figure 2). 

Mapping these putative TMPRSS2 cleavage sites in the S1 and S2′ sequences in Table 1 (colored in purple for the S1 sequences) revealed that SARS-CoV2, in contrast with other strains, acquired both a furin and an additional TMPRSS2 efficient cleavage site. Indeed, trypsin (40% identity with TMPRSS2 in the catalytic domain and 90% identical in their binding sites) efficiently cleaves both the SARS-CoV2 S1 and S2′ peptides, although the first is more rapidly processed, likely due to the RR pair in its sequence (Figure S12; Supplementary Materials). These observations suggest that both proteases can efficiently and uniquely cleave the S1 site of the highly pathogenic SARS-CoV2. Because TMPRSS2 is abundant in the lungs while furin is more ubiquitously expressed in other organs, we speculate that the increased pathogenicity of SARS-CoV2 may be due to this acquired increased tropism. However, furin is also present intracellularly and may contribute to cleaving the S1 site at the viral egress. This could contribute to the delivery of already pre-primed viral particles that could then more readily infect other cells and organs and/or spread more easily from host to host. 

### 2.2. Cellular and In Vivo Inhibition of Priming Using a Model System

Using the three-dimensional structure of furin in complex with the irreversible inhibitor dec-RVKR-CMK we derived a reversible pan-serine protease inhibitor, compound **1**, (Figure 2A) that can potently inhibit furin (IC_50_ 9 nM, Figure 2B) and hPC1/3 (IC_50_ 3 nM) (Supplementary Materials). Given that hPC1/3 and furin share similar cleavage preferences [9], it may be advantageous, if not necessary, for any inhibitor to target both human proteases. Compound **1** is also likely a moderate inhibitor of TMPRSS2 simply based on its RR mimicking motif (Figure 2). Using trypsin as model enzyme (40% identity with TMPRSS2 in the catalytic domain and >90% identical in their binding sites), compound **1** can inhibit this enzyme at 10 µM (Figure 2). Indeed, a cell-cell fusion assay with MERS-CoV strongly suggested that dec-RVKR-CMK also targeted TMPRSS2 [13], corroborating that compound **1** is potentially a pan-active serine-protease inhibitor targeting furin-like and, to a lesser extent, also TMPRSS2-like proteases. Conversely, the potent and irreversible TMPRSS2 inhibitor camostat is a poor furin inhibitor (Figure 2F). 

Similar to LF/PA anthrax toxin, SARS-CoV infects macrophages, as well as the airway epithelium [26]. Hence, we monitored the ability of compound **1** to protect RAW macrophages from LF/PA-induced cell death. The agent protected RAW macrophages in a dose-dependent manner with EC_50_ in the low micromolar range (Figure 2C) indicating a robust cellular inhibition of various possible PA-activating serine proteases, including furin and, potentially, also TMPRSS2. 

We then conducted preliminary ADME studies to assess the drug-like properties of selected agents to anticipate their use in vivo. To this end, we measured solubility (in PBS buffer, pH = 7.2, T = 25 °C, compound **1** is soluble at concentrations greater than 1 mM), plasma stability (>86% intact after 60 min incubation), and cell permeability (LogPe = −5.7; PAMPA method) (Figures S5 and S6; Supplementary Materials).

**Figure 3:**
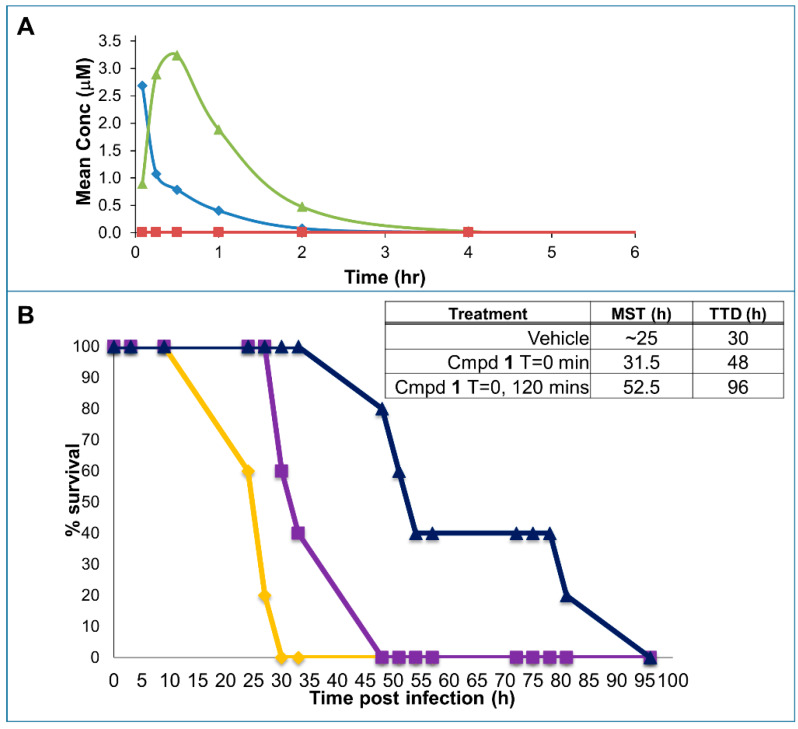
In vivo pharmacology and efficacy of compound **1**. (**A**) Preliminary pharmacokinetics (PK) studies in mice with compound **1**. Groups of 3 mice each received compound **1** via the tail veil (I.V.) (1 mg/kg, blue), intraperitoneally (I.P.) (3 mg/kg, green), or orally (P.O.) (30 mg/kg, not shown as values were too low). Blood was collected at times 5 min, 15 min, 30 min, 60 min, 2 h, 4 h, 8 h. Mean AUC I.V. = 870 h × ng/mL; Mean AUC I.P. = 2600 h × ng/mL (%F I.P./I.V. ~100%). Mean AUC P.O. = 26 h × ng/mL (%F P.O./I.V. < 1%). As a reference, levels of drug necessary to attain 50% furin inhibition is also indicated (red). (**B**) Efficacy of compound **1** in the anthrax toxemia model. With cohorts of 5 mice per group, all received 100 µg LF and 100 µg PA I.V. at time 0. Furin inhibitor compound **1** (3 mg/kg) was administered I.P. either 15 min prior to the toxin (purple curve) or 15 min before and 1.75 h after the toxin (dark blue curve) and compared to vehicle control (orange curve).

Preliminary pharmacokinetics (PK) studies in mice were also conducted (Figure 3A). In these experiments, plasma concentration of compound **1** was monitored over time after its administration via the tail veil (I.V.; 1 mg/kg), intraperitoneally (I.P.; 3 mg/kg), and orally (P.O.; 30 mg/kg). The compound is nearly 100% bioavailable when administered I.P., while insignificant adsorption is observed after oral administration (Figure 3A). However, both the I.V. and the I.P. doses reached plasma levels of the drug that are orders of magnitude greater than the IC_50_ for furin inhibition in vitro (Figure 2). Therefore, we opted to use the drug I.P. in subsequent efficacy studies.

The experimental design for the anthrax toxemia model was adopted from previous studies [31]. Here, a lethal dose of LF (100 µg) and PA (100 µg) anthrax toxins is administered I.V. in Balb/C mice. Using the toxemia rescue model [31], we designed an experiment in which groups were also injected with either a single dose 3mg/kg (I.P.) of compound **1**, or two doses spaced by 2 h, or with vehicle control. All mice in the untreated group perished in roughly 33 h, in agreement with the published studies with this model, while a remarkable and significant increase in both median survival time (MST) and time to death (TTD) was observed in both groups treated with compound **1** (Figure 3B).

## 3. Discussion

Furin and related PCs (PC2, PC1/3, PC4, PACE4, PC5/6, and PC7) are specialized serine endoproteases that cleave the multibasic motifs **R**-X-(**R**/K/X)-**R**↓(**S**)(V/A/L) [9,10,11]. In addition to its normal cellular functions, furin is also implicated in many pathogenic states. Thus, furin cleaves to maturity membrane fusion proteins of viruses and pro-toxins of a variety of bacteria, including anthrax and botulinum toxins, influenza, measles, flaviviruses and many others [9,32]. Acquisition of furin-like priming sequences correlates with increased virulence and pathogenicity. For example, the acquisition of a furin cleavage site in the priming site of the viral protein hemagglutinin (HA), necessary for influenza virus entry, is associated with the increased pathogenicity of the avian influenza viruses [33]. Perhaps more interestingly, such evolution of the influenza virus to contain furin-like sequences can be induced by repeated passages in cell culture or through animals [34].

Furin-like sequences that may contribute to increased virulence have also been identified in the coronavirus spike glycoproteins (Table 1) [1,2,3,8,12,35]. The complex mechanism of viral fusion in coronaviruses is not fully understood, but it likely comprises a first cleavage of the S1 site that allows the S2 subunit to more easily dissociate from the S1 subunit (Figure 1). The S2 subunit contains a fusion peptide, an internal fusion peptide, two heptad-repeat domains, and a transmembrane domain (Figure 1). The spike protein S1 attaches the virion to the cell membrane by interacting with its host receptor, thus initiating the infection. This occurs most likely by binding to the ACE2 receptor causing internalization of the virus into the endosomes of the host cell. Proteolysis by serine proteases of the S1 site (or by cathepsin L, in the adjacent S2 cleavage site) [8] may unmask the fusion peptide and activate membrane fusion within the endosomes. This step seems to require an additional cleavage at the S2′ site to unmask the internal fusion peptide in the S2 viral fusion protein.

However, while the S2 site is conserved among various coronavirus strains, the S1 site in SARS-CoV2 contains a furin cleavage site (Table 1). The S1 sequence is located in an exposed unstructured loop in the structure of the SARS-CoV2 spike protein (Figure 1A,B). As a result, no electron density was observed in this loop region in the recently reported Cryo-EM structure [1]. Most intriguingly, the unusual SARS-CoV2 S1 site may have also acquired an increased cleavage propensity for TMPRSS2 (Table 1) (Supplementary Materials). This may explain why TMPRSS2 appeared more important than furin for the entry of the surrogate SARS-CoV2 viral particles in cell [4]. However, while TMPRSS2 is abundant in the respiratory tract, furin is more ubiquitously found in many other organs (https://www.proteinatlas.org/ENSG00000140564-FURIN/tissue); hence, acquisition of a furin cleavage site most likely increases the tropism and the pathogenicity of the strain. Furthermore, because furin is localized in the trans-Golgi network and cycles between the trans-Golgi and the cell surface, furin cleavage in the spike protein may occur also during viral egress from the infected cells. As a result, pre-primed viral particles may be more ready to enter and infect other cell types and/or to spread among hosts. 

The common mechanisms of cell trafficking mediated by furin cleavage by both viral fusion proteins and bacterial toxins is striking. For example, anthrax toxin, similar to SARS-CoV2, requires processing of the PA sequence RKKRST (Table 1) to chaperone the internalization of the LF toxin into macrophages. Intriguingly, much like the S1 of SARS-CoV2, the PA cleavage site also contains both furin-like and TMPRSS2-like proteases recognition sites (Table 1), and it also invades macrophages, making a potentially good model system to study inhibition of priming in vivo. Intact toxins, like viral proteins, are incapable of accomplishing these processes in absence of proper priming by the host proteases. Hence, while it must be emphasized that the detailed molecular mechanisms of the spike glycoprotein-mediated viral fusion [8] and of the PA-mediated LF entry are fairly different [14,15,16,17,18,19,20,21,22,23,24,25]; both end processes depend on the activity of these priming enzymes. Nonetheless, further viral replication experiments with live SARS-CoV2 will be required for a full understanding of the potential of this approach. In recent years, several reports emerged describing improved furin inhibitors [36,37,38,39,40]. Here, we evaluate the potential of systemic administration of a furin protease inhibitor to prevent priming, using the serine protease dependent anthrax toxin as a model system. 

When tested in cell, the pan-active compound **1** was efficacious in protecting RAW macrophages from anthrax toxin (Figure 2), suggesting that the prototype agent possesses favorable pharmacological properties for in vivo studies. Because the toxicity in vivo of LF/PA toxin intimately depends on PA cleavage by serine proteases [31], this model is ideal for evaluating the inhibition of priming in vivo. Hence, Balb/C mice receiving a mixture of LF and PA (100 µg via the tail vein) were injected with either a single dose of 3 mg/kg (I.P.) of compound **1**, or two doses spaced by 2 h, or with vehicle control. According to our pharmacokinetics studies, these doses should reach blood levels of the drug sufficient to inhibit furin effectively (Figure 2B). Based on previous studies [31], mice treated with such a lethal dose of toxin die by roughly 48 h post treatment depending on the LF and PA lots and the age and strain of mice and their weight. A potent direct LF inhibitor given at 30 mg/kg I.P. 3 times a day was reported to prevent death of mice at 48 h, while no survivors were present in the control group [41]. However, no information was provided in the literature on the fate of treated mice after 48 h (no time to death was reported). In our experiments, all mice in the untreated group died by time = 33 h, in close agreement with the published studies, while a remarkable and significant increase of both median survival time (MST) and time to death (TTD) was observed in both groups treated with compound **1** (Figure 3B) even at the single dose of 3 mg/kg. 

These data clearly suggest that at least for anthrax toxin and likely for other pathogens including SARS-CoV2, furin-targeting pan protease inhibitors could be used as antiviral agents or be deployed prophylactically in emergency medicine in case of pandemic outbreaks in patients that are suspected or at risk of viral infection. Systemic administration of the inhibitors in the treated group was tolerated by mice at 3 mg/kg doses, but a maximum tolerated dose of about 10–15 mg/kg was observed in separate toxicity studies, suggesting that more targeted delivery strategies may improve the observed therapeutic window. Similar to other antiviral drugs such as Zanamivir, this could be perhaps simply accomplished by devising proper inhalable formulations, which should be facilitated by the high aqueous solubility of the agents (>1 mM). 

Recently, camostat mesylate, a covalent TMPRSS2 inhibitor already clinically approved for other indications in Japan (Figure 2D), has been proposed given that it partially blocked viral entry in surrogate cellular assays [4]. However, we found that camostat mesylate did not appreciably inhibit furin (Figure 2E), and while it may attenuate entry at relatively high concentrations (10–50 µM) [4], in our opinion it would do little to prevent furin-mediated egress of partially primed (at S1 site), hence more virulent, SARS-CoV2 viral particles.

Hence, while we await for the results of the efficacy of camostat in a very recently initiated human clinical trial with COVID19 patients (https://clinicaltrials.gov/ct2/show/NCT04321096), this report wishes to incentivize once again private and public efforts to consider developing new pan-serine protease inhibitors, perhaps taking advantage of several agents already being reported in pre-clinical studies [36,37,38,39,40], into emergency therapeutics to combat the new coronavirus SARS-CoV2 and to ward off future similar pandemics that are likely to occur when pathogens acquire the further optimized furin cleavage sites within their priming entry mechanisms.

These development efforts are particularly significant especially for coronaviruses as no viable treatments or vaccines are currently available, and at the same time other future furin-like cross-species transmission in coronaviruses seems likely. Mutations of the cleavage site in either S1 or S2′ of coronavirus strains’ spike glycoprotein can be correlated with pathogenicity, increased tropism, and crossing zoonotic barriers. Unfortunately, one could envision several mutations in SARS-CoV2 (or any other coronavirus strain) that could transform these sequences into more efficient furin and/or dual furin and TMPRSS2 cleavable sites, hence increasing their pathogenicity, virulence, and potential for spread. 

## 4. Materials and Methods

### 4.1. In Vitro Studies

Inhibition of furin and hPC1/3 activity was measured in triplicate on Corning 3676 black 384-well assay plates by using a total volume of 20 µL and the following buffer conditions: 100 mM Hepes, 2 mM CaCl_2_, pH = 7.5. Pyr-RTKR-AMC (Peptide Institute, Inc., Osaka, Japan; catalog # 3159-v) was used as fluorescent substrate at 7 µM, while the furin concentration was 80 nM per reaction well. The steady-state rate of substrate hydrolysis was monitored continuously (excitation/emission wavelengths at 365/460 nm) at 25 °C using a VictorTMX5 plate reader (PerkinElmer, Waltham, MA, USA). Inhibition of trypsin from bovine pancreas was measured in triplicate on Corning 3676 black 384-well assay plates using a total volume of 20 µL and the following buffer conditions: 100 mM Hepes, 2 mM CaCl_2_, pH = 7.5. Pyr-RTKR-AMC (Peptide Institute, Inc., catalog # 3159-v) was used as fluorescent substrate at 7 µM, while the trypsin concentration was 10 nM per reaction well. Data analysis and curve fitting for all the enzymatic assays were performed using GraphPad Prism (version 4 for Windows, La Jolla, USA). Peptides and compound **1** were synthesized using standard solid phase peptide chemistry procedures and purified using HPLC at 95% purity (Figures S1 and S8; Supplementary Materials). Camostat (mesylate) was purchased from Cayman Chemicals (Ann Arbor, USA).

Cleavage of selected coronavirus spike glyocoprotein S1 and S2′ sequences was also monitored using 1D ^1^H NMR spectroscopy of each peptide (10 µM) in the presence of furin (100 nM) after 10 min or 2 h incubation (Figures S11 and S12; Supplementary Materials). Spectra were collected on a 700 MHz Avance NMR instrument (Bruker, Karlsruhe, Germany) equipped with a TCI cryoprobe. 

In vitro metabolic stability of compound **1** was obtained by determining the compound concentration remaining over time, as determined by LCMS, after incubation with rat plasma (60 min). Cell permeability was assessed using the PAMPA (parallel artificial membrane permeability assay) method. Values reported are the permeability rate (LogPe) calculated using the following equation:

LogPe = log{C × ln(1 − [drug]Acceptor/[drug] equilibrium)}C = (VD × VA)/((VD + VA) Area × time))(1)

### 4.2. Macrophage Protection from Anthrax Toxin (LF/PA)

To assess whether compound **1** prevented anthrax LF/PA toxin entry we used RAW 264.7 murine monocyte macrophages (4.5 × 10^4^ cells/well) in 96-well tissue culture plates. Cells were grown in Hyclone DMEM (4500 mg/L Glucose, 110 g/L Sodium Pyruvate) and supplemented with 5% fetal bovine serum, 2 mM Glutamax (Invitrogen, Carlsbad, CA), and 1% penicillin/streptomycin (Omega Scientific). RAW 264.7 murine monocyte macrophage cells were cultured at 37 °C in a humidified incubator containing 5% CO_2_, overnight. Subsequently, cells were replenished with fresh serum-free medium (0.1 mL/well) and treated with a pre-incubated solution of test compounds (at various doses: 0.015 µM to 33.3 µM) and anthrax toxin consisting of LF (37.5 ng/mL) and PA_83_ (500 ng/mL). Cell viability was assessed (ATPlite, Perkin Elmer, Waltham, MA), after incubation for 3.5 h (T = 37 °C), and normalized to control treatments (1% DMSO only, and LF/PA only).

### 4.3. Pharmacokinetics and Toxicity Studies

To assess compound **1** toxicity, 8 week old female Balb/c mice of 20 g as average weight (strain code: 490, Charles River) received I.P. injections according to the following doses and regimen: 3 mg/kg q.i.d every 2 h for 1 or 2 days; 6 mg/kg t.i.d every 3 h for 1 or 2 days; 10 mg/kg b.i.d every 6 h for 1 day; a single dose of 15 mg/kg and 20 mg/kg. Each group contained 3 mice that received either vehicle (PBS, pH = 7.4) or test compound in a 200 µL final volume. Of the mice that received the highest dose (20 mg/kg), 2 out of 3 died 15 min after compound injection, and all mice which received a 15 mg/kg dose died 1 h after compound administration. Therefore, the maximum tolerated dose (MTD) was ~10 mg/kg b.i.d. Acute toxicity was also examined using blood chemistry analyses 5 min after a single high dose injection (20 mg/kg). From each mouse, 400 μL of whole blood was collected in lithium heparin tubes. Tubes were inverted to mix blood and anti-coagulant and immediately centrifuged at 2500 rpm for 10 min. Plasma (100 µL) was collected and analyzed on a Vetscan VS2 Instrument (Abaxis Veterinary Diagnostic) using comprehensive diagnostic rotors. Compared to the control group, compound treated mice showed gross hemolysis, while other blood chemistry values were normal. PK studies were conducted by Agilux (Cambridge, MA) as described in the manuscript and in the caption to Figure 3.

### 4.4. Anthrax Toxemia Model

Eight week old female Balb/c mice (average body weight ~20 g, (strain code: 490, Charles River), received I.V. co-injections of 100 µg of recombinant lethal factor (LF, Lists laboratories, catalog number 172) and 100 µg of recombinant protective antigen (PA, Lists laboratories, catalog number 171). The control group received anthrax toxin only while test groups received compound **1** at different times: one dose of 3 mg/kg at 15 min before toxin injection for the first group, and two doses of 3 mg/kg at 15 min before toxin injection and 1.75 h after toxin injection, for the second group. Compound **1** was administered I.P. as PBS solution (pH = 7.4) in 200 µL as final injection volume. Mice body weight was recorded every day and animals were observed multiple times daily for vital signs and distress. Our animal research was carried out using IACUC (Institutional Animal Care and Use Committee) approved protocols at the Sanford Burnham Prebys Medical Discovery Institute Animal Facility. The facility is accredited by AAALAC International (the Association for Assessment and Accreditation of Laboratory Animal Care International. Participating in the AAALAC accreditation program and meeting their rigorous standards demonstrates our commitment to humane and responsible animal research and our dedication to good science.

At the end of the experiments, animals were euthanized using CO_2_ asphyxiation following National Institutes of Health guidelines for the care and use of laboratory animals (National Research Council, 1996) and according to the IACUC approved protocol at the Sanford Burnham Prebys Medical Discovery Institute Animal Facility.

## Figures and Tables

**Table 1:** Examined S1 and S2′ cleavage sites in selected coronavirus strains. In bold are residues that are preferred by furin-like proteases. In S1 and S2′ sequences, purple residues indicate preferred TMPRSS2 cleavage preferences: R↓K > R↓R >> R↓S. A score value indicative of furin cleavage preference for each motif is also reported.

*Coronavirus*	S1 Site	Score	S2′ Site	Score
Bat RaTG13	TQTNS**R**↓**SV**	0.18	SKPS**KR**↓**S**F	0.42
Bat ZC45/Bat ZXC21	TASIL**R**↓**S**T	0.12	SKPS**KR**↓**S**F	0.42
SARS CoV	TVSLL**R**↓**S**T	0.10	LKPT**KR**↓**S**F	0.31
MERS-CoV	TP**R**SV**R**↓**SV**	**0.60**	GS**R**SA**R**↓**SV**	**0.77**
SARS-CoV2	SP**R**RA**R**↓**SV**	**0.71**	SKPS**KR**↓**S**F	0.42
***Bacterial toxin***				
*B. anthracis PA*	NS**R**K**KR**↓**S**T	**0.88**		

## Supplementary Materials

The following are available online at https://www.mdpi.com/1420-3049/25/10/2424/s1: Figure S1: Synthetic scheme for compound **1**, Figure S2: HPLC profile of compound **1**, Figure S3: MS profile of compound **1**, Figure S4: ^1^H NMR spectrum of compound **1** in d6-DMSO, Figure S5: Plasma stability of compound **1**, Figure S6: Microsomal stability of compound **1**, Figure S7: Compound **5** synthetic scheme and general description, Figure S8: HPLC profile of compound **5**, Figure S9: MS profile of compound **5**, Figure S10: ^1^H NMR spectrum of compound **5** in d6-DMSO, Figure S11: NMR-detected cleavage of selected S1 and S2′ loops of coronavirus strains by furin, Figure S12: NMR-detected cleavage of selected S1 loops of coronavirus strains by trypsin. Table S1: Chemical structures of selected synthesized compounds and relative critical measurements to assess their drug-like characteristics, Table S2: Specificity of selected inhibitors for furin-like PCs.
