# Supplementary material for: Potential Therapeutic Targeting of Coronavirus Spike Glycoprotein Priming
Source: Molecules. 2020 May 22;25(10):2424. doi: 10.3390/molecules25102424 (PMC7287953; doi:10.3390/molecules25102424)
Supplement: Supplementary file 1 [file molecules-25-02424-s001.pdf]

Article

Potential Therapeutic Targeting of Coronavirus Spike Glycoprotein Priming

Elisa Barile [1,2], Carlo Baggio [1], Luca Gambini [1], Sergey A. Shiryaev [3], Alex Y. Strongin [3] and Maurizio Pellecchia [1,*]

1 Division of Biomedical Sciences, School of Medicine, University of California Riverside, Riverside, CA 92521, USA; elisabarile@gmail.com (E.B.); carlo.baggio@medsch.ucr.edu (C.B.); lucaga@ucr.edu (L.G.)
2 Current address: Takeda California, Inc., San Diego, CA 92121, USA
3 Sanford Burnham Prebys Medical Discovery Institute, La Jolla, CA 92037, USA; shiryaev@sbpdiscovery.org (S.S.); strongin@sbpdiscovery.org (A.S.)
* Correspondence: maurizio.pellecchia@ucr.edu

Contents

Figure S1: Synthetic scheme for compound 1
Figure S2: HPLC profile of compound 1
Figure S3. MS profile of compound1
Figure S4. ^{1}H NMR spectrum of compound 1 in d_6-DMSO
Figure S5. Plasma stability of compound 1
Figure S6. Microsomal stability of compound 1
Figure S7. Compound 5 Synthetic scheme and general description
Figure S8. HPLC profile of compound 5
Figure S9. MS profile of compound 5
Figure S10. ^{1}H NMR spectrum of compound 5 in d_6-DMSO
Figure S11. NMR detected cleavage of selected S1 and S2′ loops of coronavirus strains by furin.
Figure S12. NMR detected cleavage of selected S1 loops of coronavirus strains by trypsin.
Table S1 Chemical structures of selected synthesized compounds and relative critical measurements to assess their drug-like characteristics
Table S2 Specificity of selected inhibitors for Furin-like PCs

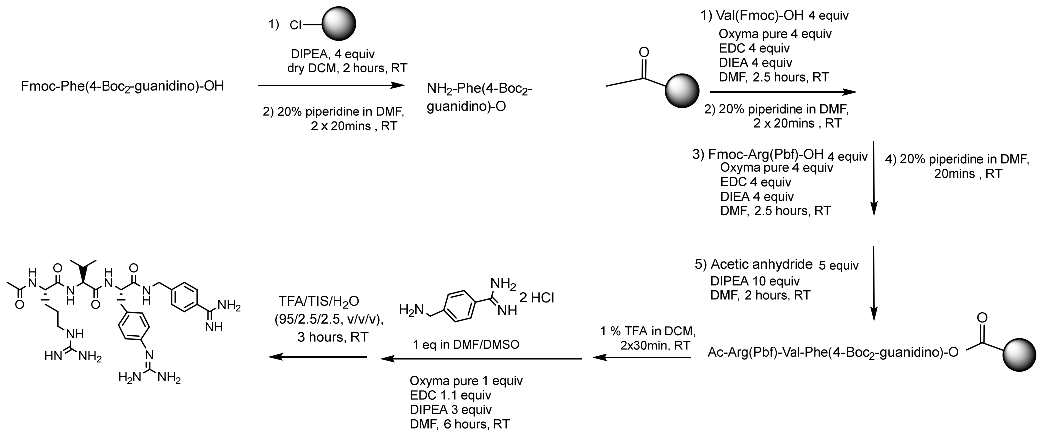

Figure S1. Synthesis of compound 1 and analogs. Compound **1** and analogs (**2**-**3**) were prepared by a combination of solid phase and solution synthesis. Briefly, the N-acylated segment P2-P4 protected in P2 and P4 positions, after weak acidic cleavage from the 2-chloro-tritylchloride resin was purified and coupled to unprotected 4-amidinobenzylamine and derivatives, followed by side chains deprotection. All the final compounds were purified by both preparative and semipreparative reverse phase HPLC, lyophilized and obtained as TFA or HCl salts.

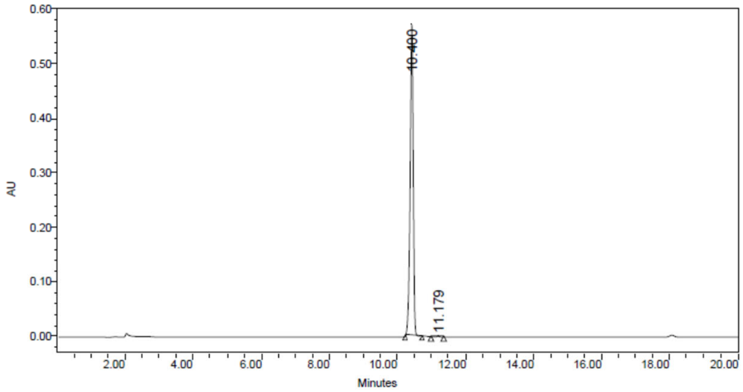

	RT (min)	Area (µV*sec)	% Area	Height (µV)	% Height
1	10.400	3615078	99.84	567018	99.79
2	11.179	5907	0.16	1203	0.21

Figure S2. HPLC profile of compound **1**.

Figure S3. MS profile of compound 1

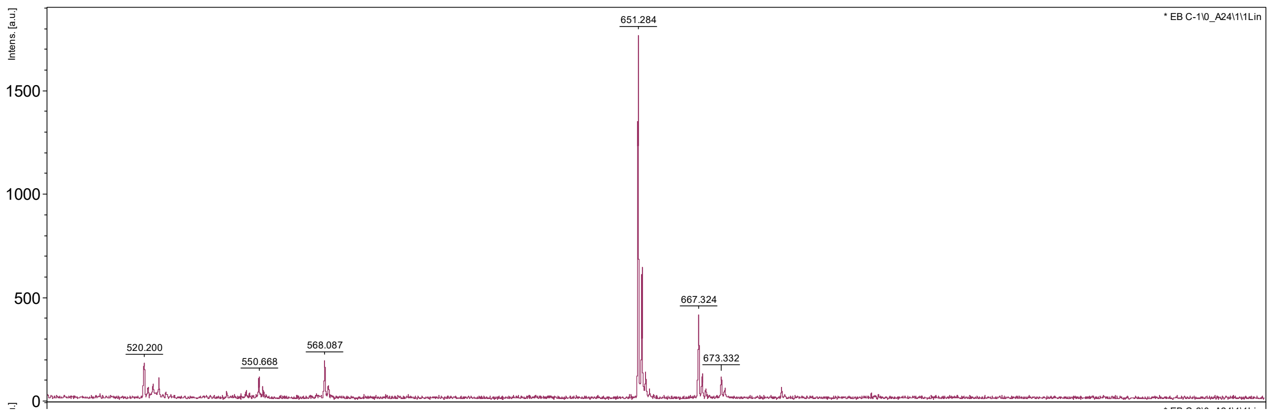

Figure S4. ^{1}H NMR spectrum of compound 1 in d_6-DMSO**.**

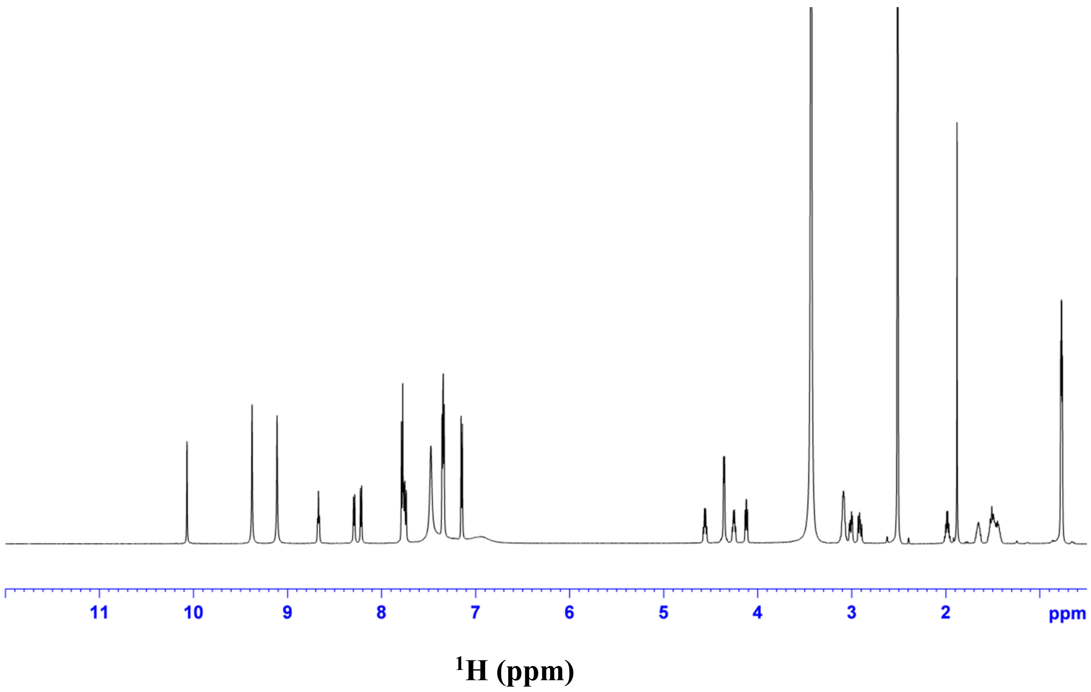

Figure S5. Plasma stability analysis of compound 1

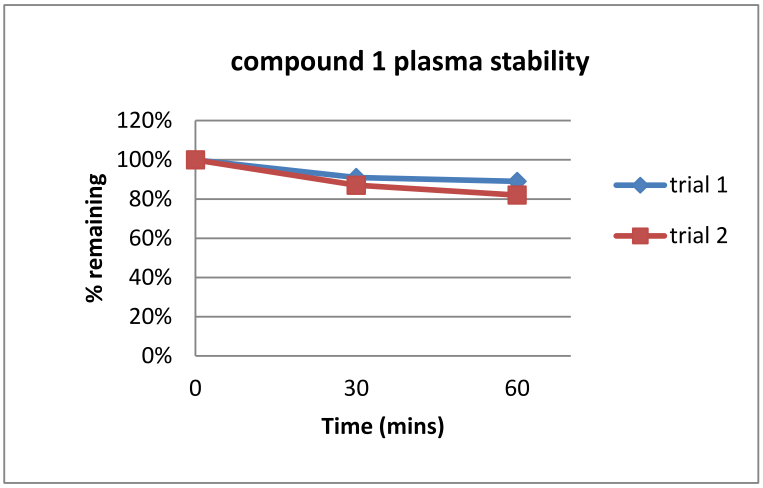

Figure S6. Microsomal stability analysis of compound 1

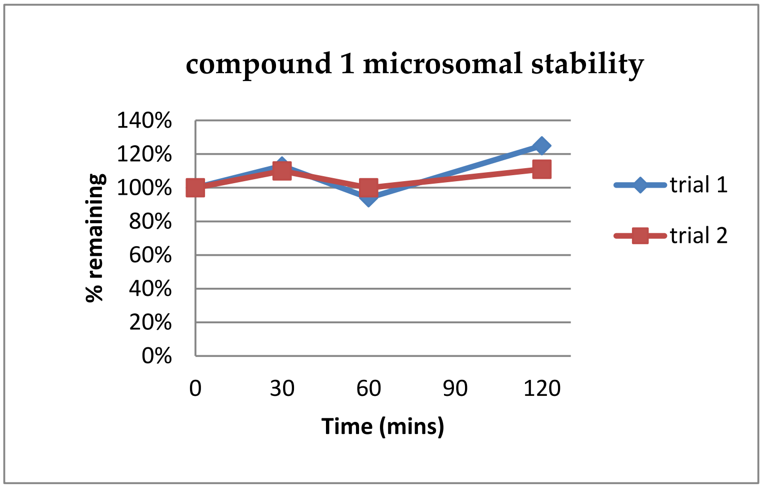

Figure S7. Synthesis of compound 5. Compound **5** and analogs (**4** and **6**) were prepared by a combination of solid phase and solution synthesis. Briefly, the segment P2-P4 protected in P2, after weak acidic cleavage from the 2-chloro-tritylchloride resin was coupled to unprotected 4-amidinobenzylamine, followed by final side chain deprotection. All the final compounds were purified by both preparative and semipreparative reversed phase HPLC and obtained as lyophilized powder.

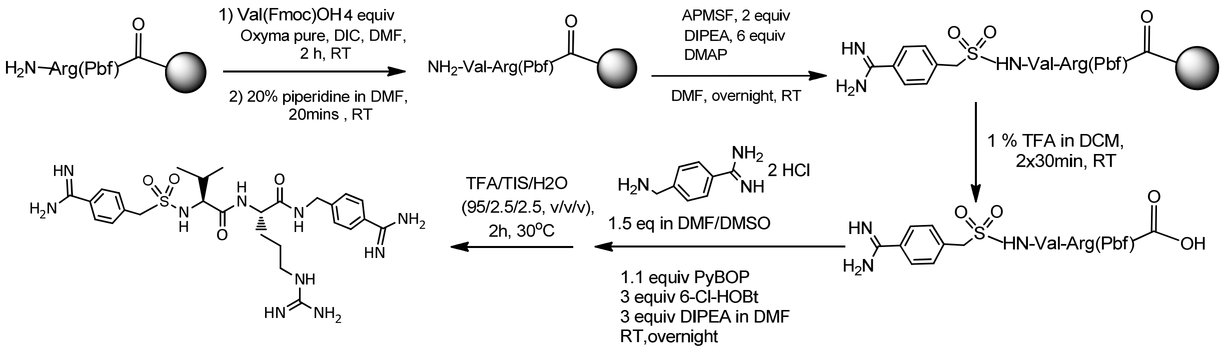

Purity of all compounds was obtained by analytical HPLC on a Breeze system from Waters Co. using a 5◎m, 4.6 x 150 mm symmetry reverse phase column with a linear gradient of acetonitrile containing 0.1% TFA at a flow rate of 1 mL/min and by ^{1}H NMR spectra recorded on a Bruker 600 MHz instrument

Figure S8. HPLC profile of Compound 5

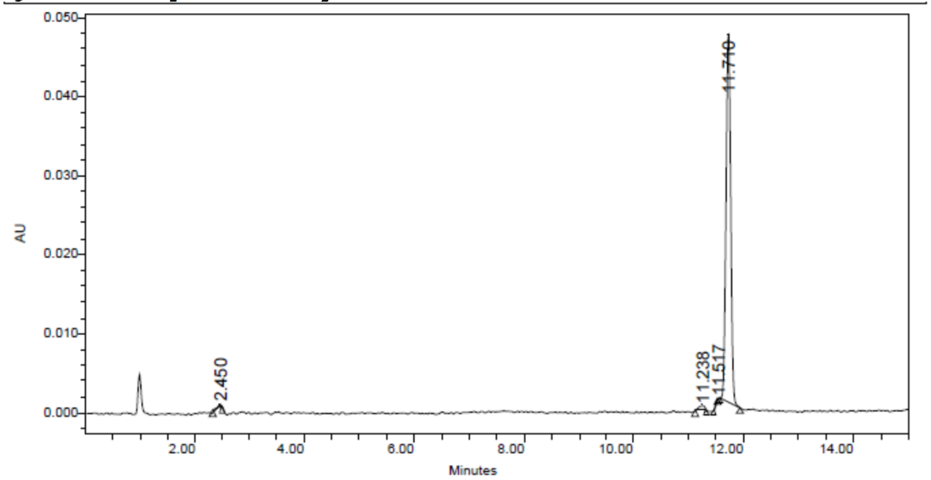

	RT (min)	Area (⍞V*sec)	% Area	Height (⍞V)	% Height
1	2.450	2776	0.98	530	1.10
2	11.238	4406	1.55	580	1.21
3	11.517	224	0.08	92	0.19
4	11.710	277095	97.40	46863	97.50

Figure S9. MS profile of compound 5

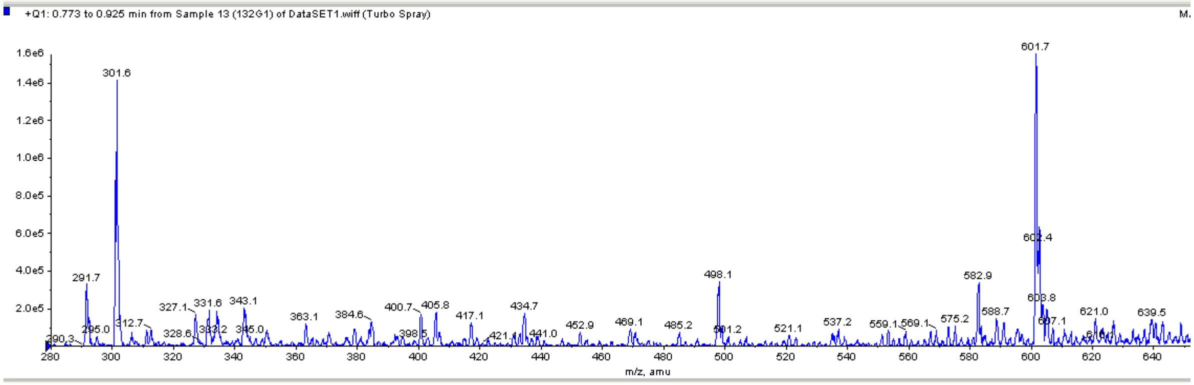

Figure S10. ^{1}H NMR spectrum of compound 5 d$_6$-DMSO.

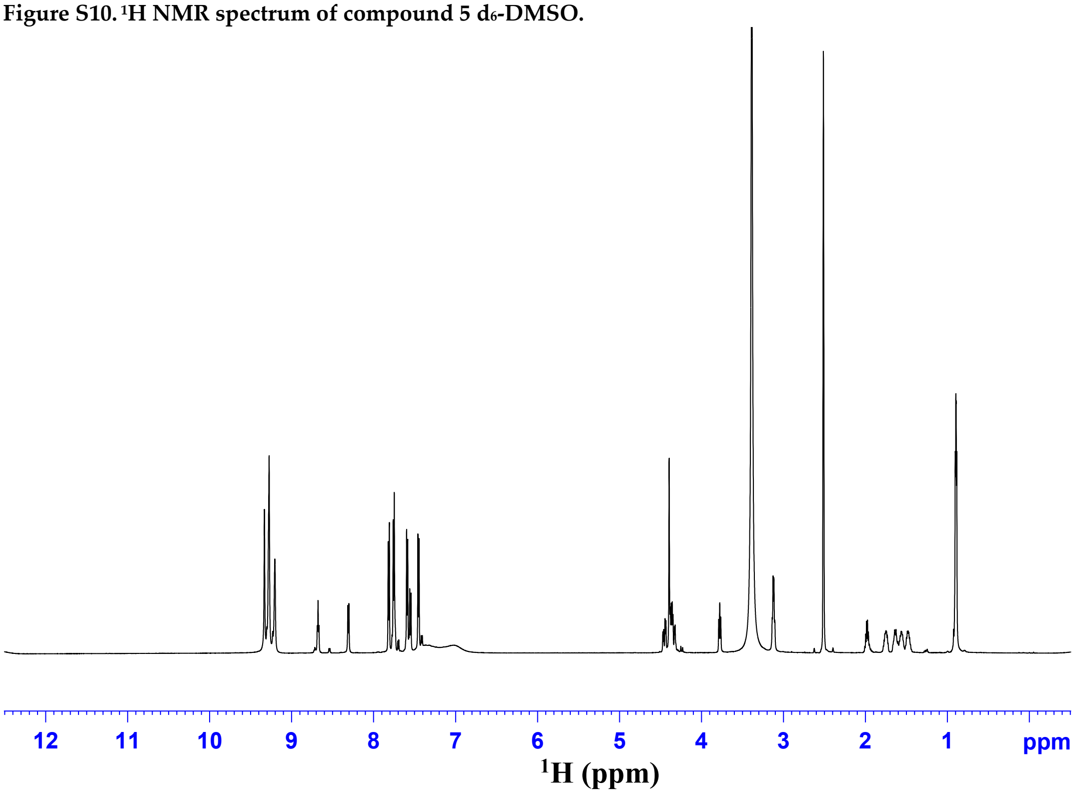

Cmpd ID	IC_{50} (μM)			
	Furin	hPC1/3	hPC2	hPC7
Compd 1	0.009	0.003	1.86	11.8
Compd 2	0.279	0.049	2.05	4.05
Compd 3	12.4	>100	7.75	16.1
Compd 5	0.338	0.014	1.17	3.19
Compd 6	12.7	0.483	>30	14.0

Compd	Structure	MW	Solubility[a]	Furin IC_{50} (μM)	Plasma stability[b]	Microsomal stability[c]	Cell permeability (PAMPA)[d]
Compd 1	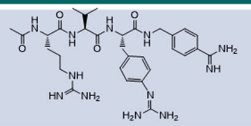	650.8	>1.0 mM	0.009	86 %	100 %	-5.7
Compd 2	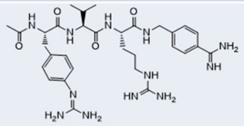	650.8	>1.0 mM	0.279	n.d.	n.d.	n.d.
Compd 3	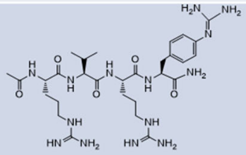	674.8	>1.0 mM	12.4	n.d.	n.d.	n.d.
Compd 4	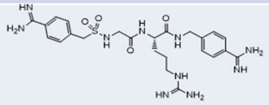	558.7	>1.0 mM	8.02	n.d.	n.d.	n.d.
Compd 5	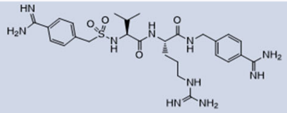	600.7	>1.0 mM	0.338	83 %	100 %	-5.1
Compd 6	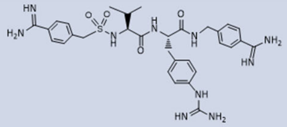	648.8	>1.0 mM	12.7	98%	100 %	-5.2

Table 1. Chemical structures of selected synthesized compounds and relative critical measurements to assess their drug-like characteristics. *a*. Solubility was assessed by 1D ^{1}H NMR experiments in PBS. *b*. Stability of the compound in rat plasma as determined by LCMS. Values indicate amount of compound remaining after 60 minutes incubation. *c*. Stability of test compound in rat microsomes. The reported value indicated the amount of compound remaining after 120 minutes incubation. *e*. Cell permeability as assessed by the PAMPA method. Values reported are the permeability rate (LogPe) calculated using the following equation: LogPe=log{C•-ln(1-[drug]Acceptor/[drug] equilibrium)}C = (VD • VA) / ((VD + VA) Area • time)).

Table S2. Specificity of inhibitors listed in Table 1 for Furin-like PCs.

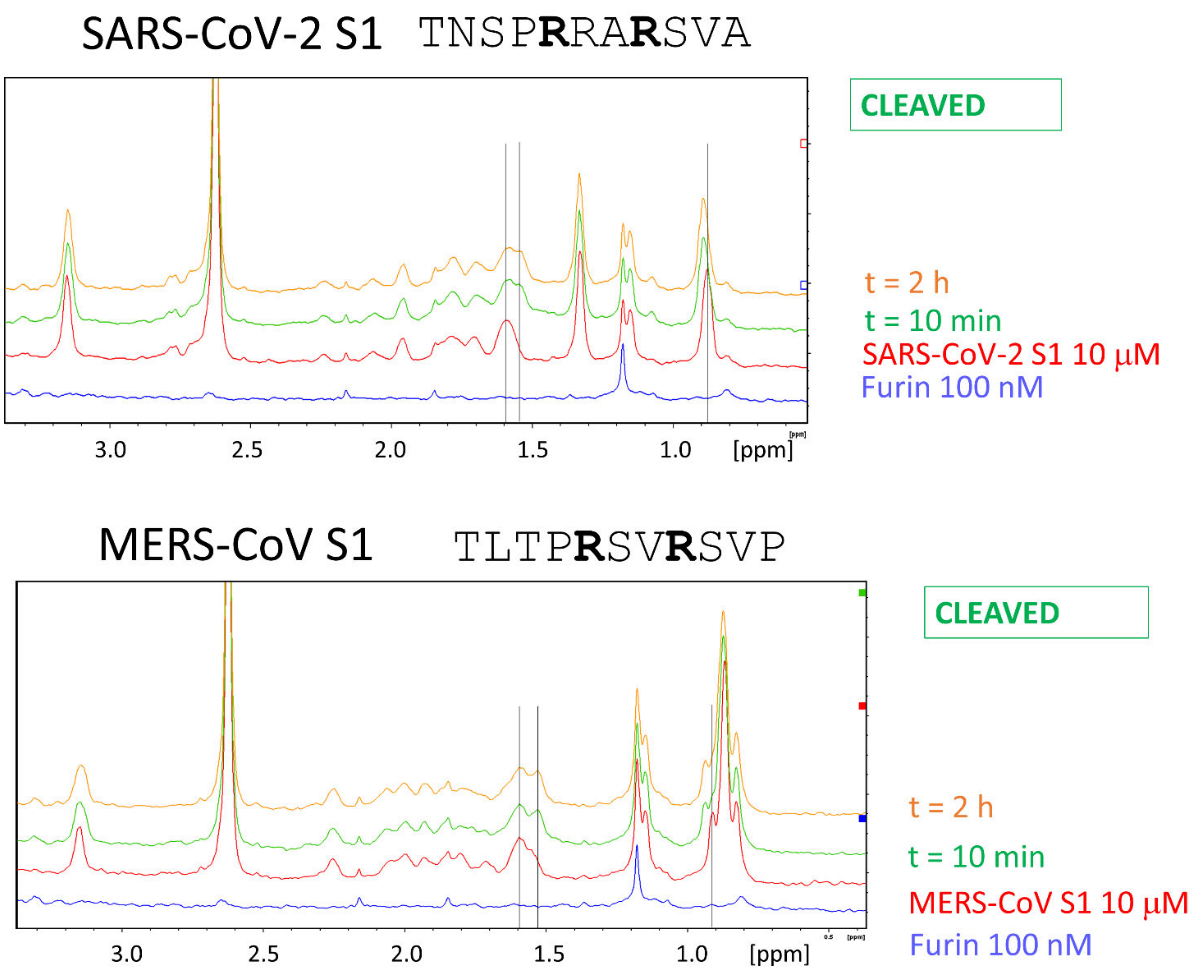

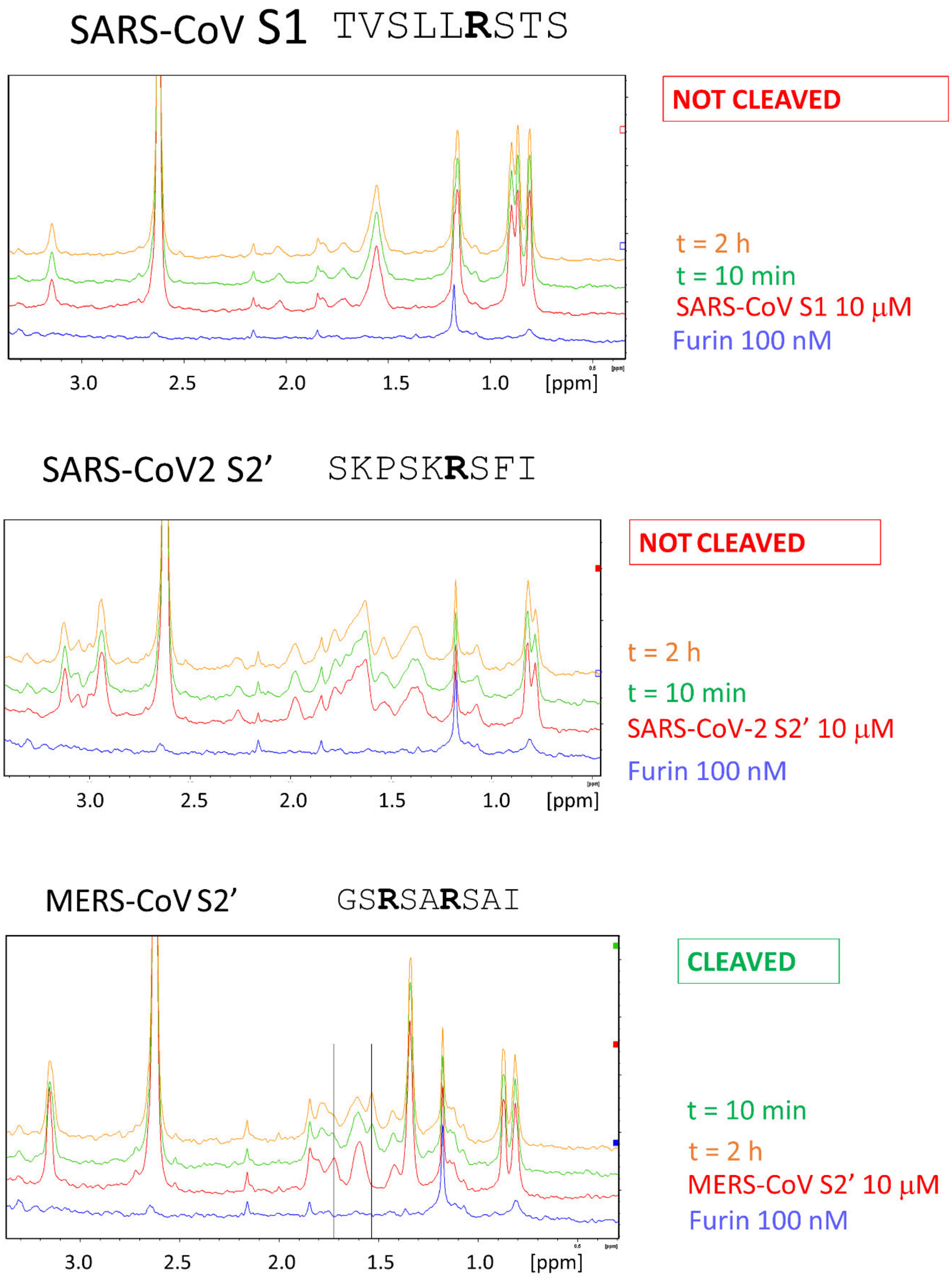

Figure S11. 1D ^{1}H NMR spectra recorded for each indicated peptides (10 ◎M in 25 mM TRIS-d11 pH 7.5, 150 mM NaCl, 2 mM CaCl2, 1% d6-DMSO) incubated with 100 nM Furin. Spectra were acquired on a 700 MHz Avance Bruker instrument equipped with a TCI-cryoprobe, operating at 700 MHz ^{1}H frequency.

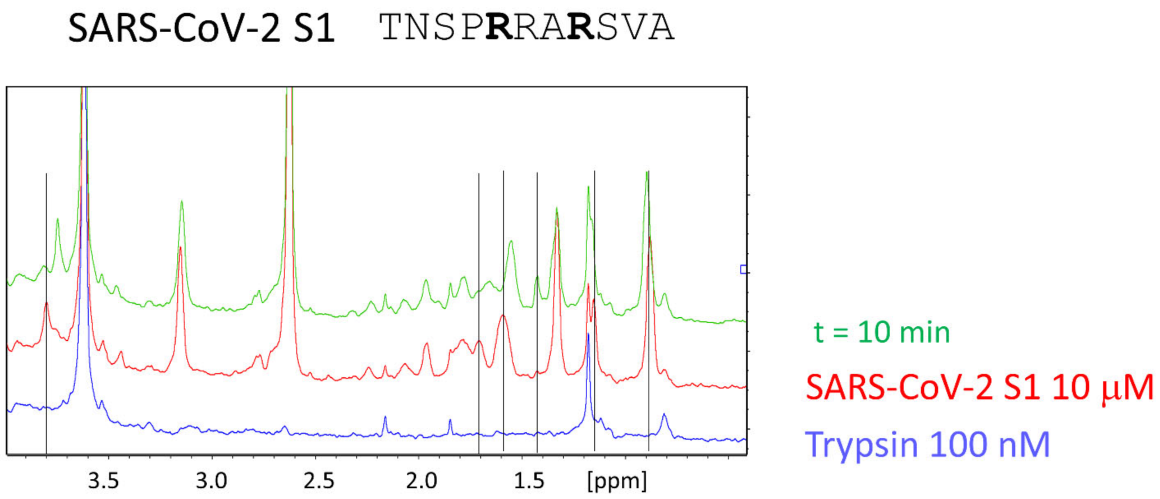

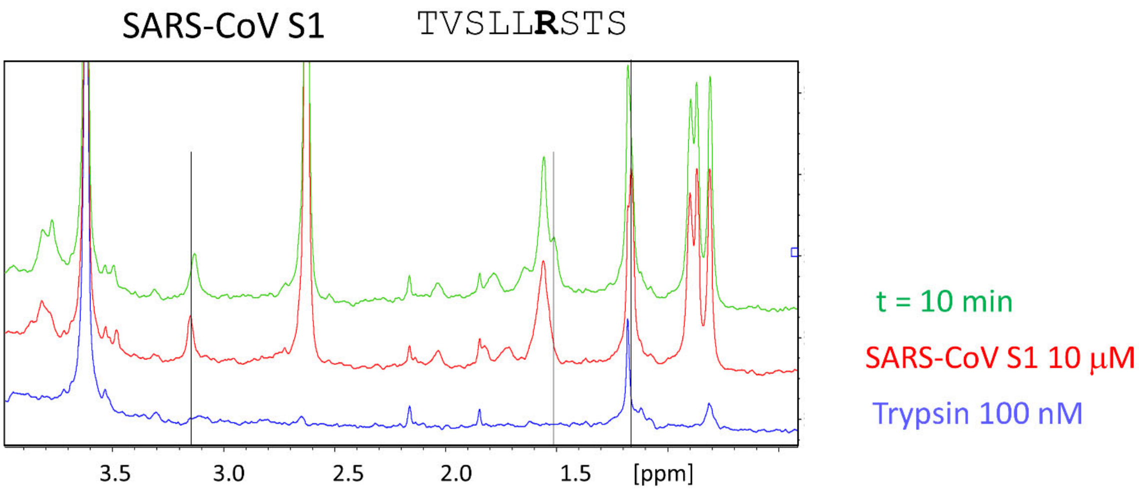

Figure S12. 1D ^{1}H NMR spectra recorded for each indicated peptides (10 μM in 25 mM TRIS-d11 pH 7.5, 150 mM NaCl, 2 mM CaCl2, 1% d6-DMSO) incubated with 100 nM trypsin. Spectra were acquired on a 700 MHz Avance Bruker instrument equipped with a TCI-cryoprobe, operating at 700 MHz ^{1}H frequency.
